# A minimally manipulated preservation and virus inactivation method for amnion/chorion

**DOI:** 10.3389/fbioe.2022.952498

**Published:** 2022-08-11

**Authors:** Shang Zhang, Lichang Gao, Pin Wang, Yuyan Ma, Xiaoliang Wang, Jie Wen, Yu Cheng, Changlin Liu, Chunxia Zhang, Changfeng Liu, Yongli Yan, Chengru Zhao

**Affiliations:** ^1^ Success Bio-Tech Co., Ltd., Biomedical Material Engineering Laboratory of Shandong Province, Jinan, China; ^2^ Department of Gynecology and Obstetrics, Qilu Hospital, Cheeloo College of Medicine, Shandong University, Jinan, China; ^3^ Liangchen Biotechnology (Suzhou) Co., Ltd., Suzhou, China

**Keywords:** diabetic foot ulcer (DFU), chronic wound, animal model, amnion, virus inactivation, growth factor (GF)

## Abstract

Allogeneic amnion tissues have been widely used in tissue repair and regeneration, especially a remarkable trend of clinical uses in chronic wound repair. The virus inactivation procedures are necessary and required to be verified for the clinical use and approval of biological products. Cobalt-60 (Co-60) or electron-beam (e-beam) is the common procedure for virus and bacterial reduction, but the excessive dose of irradiation was reported to be harmful to biological products. Herein, we present a riboflavin (RB)-ultraviolet light (UV) method for virus inactivation of amnion and chorion tissues. We used the standard *in vitro* limiting dilution assay to test the viral reduction capacity of the RB-UV method on amnion or chorion tissues loaded with four types of model viruses. We found RB-UV was a very effective procedure for inactivating viruses of amnion and chorion tissues, which could be used as a complementary method to Co-60 irradiation. In addition, we also screened the washing solutions and drying methods for the retention of growth factors.

## Introduction

Amnion is an avascular, aneural, and alymphatic tissue and has limited expression of human leukocyte antigen (HLA)-I antigens (Jirsova and Jones, 2017; Huddleston et al., 2020). The immune-privileged state makes amnions a convenient resource of valuable tissue-repairing biomaterials for allogeneic uses (Magatti et al., 2018; Zhang et al., 2018; Wassmer and Berishvili, 2020). It is almost a sustainable and limitless pool of desirable allogeneic materials for tissue repairing and regeneration because of the easy collection of amnion tissues from the birth-delivering course. Amnion and chorion mainly comprise type I collagen, adhesion proteins, traces of hormones, bioactive growth factors, cytokines, and enzymes (Huddleston et al., 2020). Amnion is the inner side layer of the placenta. A human placenta in general comprises two layers including amnion and chorion which develop from the sibling tissues, although the chorion also includes a thin layer of degenerated decidual membrane originally from the maternal tissues (Parolini et al., 2008). A part of the chorion develops and outgrows into a placenta disk which connects to the womb and helps to provide nutrition and oxygen for the sibling linked through the umbilical cord. Around the disk, the amnion and chorion layers form a sack wrapping the sibling.

As a biocompatible and bioactive allogenic tissue-repairing material, amnion has been widely used in biomedical applications, including corneal ulcers, diabetic foot ulcers, venous ulcers, postoperative dehiscence, and burn wounds (Sheikh et al., 2014; Bianchi et al., 2018; Didomenico et al., 2018; Zelen et al., 2018; Tettelbach et al., 2019; Lipovy and Forostyak, 2020; Schuerch et al., 2020). Amnion substitutes have been widely used in ophthalmology surgery for corneal protection and regeneration as common practices. They are used in various types of corneal ulcers, including chemical ulcers, bacterial infection ulcers, viral infection ulcers, and rheumatological ulcers (Kim et al., 2001; Kara, 2018; Hopkinson et al., 2020; Schuerch et al., 2020). In the last decade, amnion and chorion have been intensively used for chronic skin wounds, including diabetic foot ulcers and venous ulcers, as a skin substitute and with the function of inducing recovery and regeneration (Sheikh et al., 2014; Bianchi et al., 2018; Didomenico et al., 2018; Zelen et al., 2018; Dhall et al., 2019; Tettelbach et al., 2019; Schmiedova et al., 2021). Clinical evidence showed its proven functionality in skin regeneration. Chronic wounds treated with the amnion/chorion complex recovered much more quick compared with standard care, other biomedical materials, or even a tissue-engineered skin product (Zelen et al., 2013; Lavery et al., 2014; Zelen et al., 2015; Didomenico et al., 2018; Tettelbach et al., 2019). Amnion is often used solely or together with chorion to increase the thickness and the bioactive contents of the graft (Koob et al., 2015). The possible inside mechanisms were indicated to be the bioactive factors contained within amnion and chorion tissues, including growth factors, cytokines, chemokines, and matrix metalloproteinases (MMPs) (Choi et al., 2009; Rodriguez-Ares et al., 2009; Koob et al., 2013; Koob et al., 2014; Koob et al., 2015; Moore et al., 2020).

Currently, solely in the United States, there are more than 100 amnion or chorion products in the market (Amniochor, 2017). Most of them were regulated as “361 products” by the FDA, which mainly refers to human cells, tissues, and cellular- and tissue-based products (Fda, 2018). Some of them were licensed through premarket notification 510(k). Mimedx Ltd. is among the largest amnion/chorion providers. Its annual revenue peaked at 0.38 billion United States dollars in 2018 and remained around 0.25 billion in the last 2 years according to their full-year reports (Mimedx, 2022). Their products mainly deal with diabetic foot ulcers, venous leg ulcers, debridements, and decubitus ulcers. They have accumulatively delivered more than 2 million placental allograft tissues for clinical use, according to the official website (Mimedx, 2021).

There are three popular ways of preserving amnion or chorion tissues, including air-drying, lyophilization, and cryopreservation (Koob et al., 2013; Dhall et al., 2018; Dhall et al., 2019; Jacob et al., 2020; Mcdaniel et al., 2021). The dehydration method usually involves a drying process using a fan oven; lyophilization literally means using a freeze-dryer to dehydrate the tissue; while the cryopreservation method often involves a cryopreservation reagent such as glycerol or DMSO during a snap freezing procedure; then, the tissues are immersed in liquid nitrogen or dry ice.

Allogeneic and xenogeneic biological products usually require virus inactivation processes for clinical use and marketing approval. In the past decades, the blood-transmitted diseases such as human immunodeficiency virus (HIV) and hepatitis B virus (HBV) have been greatly reduced because more attention was paid to the risks and more sensitive detection methods were invented to screen the donor samples for blood transfusion or for making biological products (Who, 2004). The window period of pathogens during which an infectious donor cannot be detected is caused by the initial minimal replication of viruses and the time lag of acquired immunity at the beginning of infection. Due to the inevitably existing window period of virus detection, virus inactivation is still a necessary step to eliminate the like-hood of blood-transmitted diseases (Marschner and Goodrich, 2011). Plenty of data about the necessity of virus inactivation were reported for blood-derived products, which has referential values for allogeneic biomaterials. It is recommended by the WHO that viral inactivation method should be applied to all blood plasma-derived protein solutions (Who, 2004). For unscreened donors, the estimates of the frequency of occurrence in blood donors of hepatitis viruses and HIV are as follows: HBV 1/10000, hepatitis C virus (HCV), 1/50–1/100, and HIV 1/1000–1/10000 in EU and United States populations (Who, 2004). Screening by antibody tests is not really effective in reducing virus titers in blood products, because blood-origin products were often pooled; it was reported that 65 out of 85 (76%) plasma pools were still HCV PCR positive after screening by the first-generation anti-HCV antibody test, and 49 out of 123 (39%) were HCV PCR positive following second-generation antibody test (Nubling et al., 1995; Who, 2004). It is estimated that nucleic acid amplification technology could greatly reduce the window period compared with regular tests, for example, 22 days reduced to 10 days for HIV, 82 days reduced to 9 days for HCV, and 59 days reduced to 49 days for HBV (Schreiber et al., 1996; Who, 2004). However, the detection limit of screening tests and the errors in the process may still occur, particularly when the size of the donor number is large. The screening helps to minimize the risk but it is not sufficient to ensure safety in itself. The production process to remove or inactivate viruses is a crucial element.

The currently used amnion and chorion are donated placenta tissues from birth delivery. Both amnion and chorion membrane tissues are usually dissected and cut off from a placenta disk. Both layers are used either individually or in combination as a complex for tissue substitution (Koob et al., 2015). Sterilization steps are obligated to minimize the potential of carrying transmitting pathogens such as bacteria and viruses. Irradiation is overwhelmingly employed as the pathogen reduction procedure, including Cobalt-60 (Co-60) and the electron-beam (e-beam). However, it was reported that high doses of irradiations would diminish the therapeutic capacity of biological products (Grieb et al., 2002; Moore, 2012). In addition, minimal manipulation is usually required to manufacture such kinds of amnion products (Fda, 2020). For the aforementioned reasons, herein we present a method for virus inactivation of amnion products, particularly using a very low dose of irradiations; and together with the method of washing and drying, we provide a way of preserving amnion/chorion tissues.

## Materials and methods

### Tissue procurement and ethics statement

Tissue procurement and ethics statements were provided by Qilu Hospital. Tissues were collected from eligible donors after obtaining written, informed consent.

### Isolation and washing steps

Amnions and chorions from caesarean delivery were isolated as described elsewhere (Daniel et al., 2013). In brief, amnion and chorion were dissected from the placenta by scissors and separated gently with gloved hands. Amnion and chorion were washed separately in PP bottles and in a shaking incubator with pure water, normal saline, or hypertonic saline (18% NaCl in H_2_O) depending on the experiment design. Amnion was washed twice, each for 1 h, rinsed in pure water, then kept in the fridge with normal saline overnight for the following virus inactivation procedures. Chorion was washed three times, each time for 1 h, and washed once again for another 2 h with 0.5% Triton X-100 in pure water, rinsed with pure water, then kept in the fridge with 0.1% EDTA-2Na in PBS overnight for the following virus inactivation procedures.

### Riboflavin-ultraviolet light virus inactivation

RB is also known as vitamin B2 (VB2). 5 ml of 500 μmol/L RB stock solution (J&K, Riboflavin, 4469220) was diluted into 50 ml working solutions using normal saline. The washed samples described earlier were immersed within the aforementioned RB working solution for 2 h. Amnion or chorion was then spread on a tray and incubated in a UVB crosslinker (Spectronics, XL-1500B/FA, 312 nm UV light bulbs) with an energy of 3–7 J/cm^2^ as required by the experiments, the samples were turned over after each cycle of 1 J exposure.

### Drying steps

The amnion and the chorion after virus inactivation were then rinsed in pure water and dried on a patterned drying board, which was designed locally, either separately or combined as a multilayer amnion/chorion complex (Daniel et al., 2013). The air-dry was performed using a fan oven (Boxun, BGZ-240) at 37°C. The freeze-dry was performed using a −40°C freeze-dryer (Scientz, 100F) for 48 h.

### Co-60 virus inactivation

The amnion, chorion, or the dual-layer complex was then cut and packed using a blister package and a Tyvek lid for the following Co-60 irradiation. Co-60 irradiation was performed by Zhongjin Irradiation Incorporated Company. The Co-60 irradiation doses were monitored using dosimeters (Harwell Red 4034 pmma).

### ELISA and cytokine array

bFGF and HGF were detected using a Human bFGF ELISA kit (Neobioscience, EHC130.96) and a Human HGF ELISA kit (Neobioscience, EHC138.96). Experiments were performed using a standard curve method according to the user manuals. For all the tests, the standard curves reached an R-square > 0.99. Total protein was detected using a BCA kit (Beyotime, BCA Protein Assay Kit, P0011). The testing samples were cut into minimal particles and homogenized using a homogenizer (DLAB, D160) in an IP and western cell lysis buffer (Beyotime, P0013) with PMSF (Beyotime, ST506) according to the user manual, followed by the full lysis using the lysis buffer within the kit at 4°C overnight and a centrifuge process, the lysate was collected and measured using the aforementioned ELISA assay kits. The bFGF and HGF results were normalized using the total protein concentration of each sample lysate. The high-throughput multicytokine-array screening of growth factors and cytokines was performed by Raybiotech using a Human Cytokine Array Q440 kit (QAH-CAA-440).

### Virus inactivation test

The virus inactivation test was performed using a standard *in vitro* limiting dilution assay (Grieb et al., 2002). Pseudorabies virus (PRV), Sindbis virus, encephalomyocarditis virus (EMCV), and porcine parvovirus (PPV) were used as model viruses representing four types of viruses, that is, DNA enveloped, RNA enveloped, DNA non-enveloped and RNA non-enveloped. ST cells were used as the indicating cells for PRV and PPV infection and Vero cells were used for Sindbis and EMCV infection. The Spearman–Karber method was employed for TCID50 calculation (Grieb et al., 2002; Ramakrishnan, 2016). The virus load was recorded by a unit of IgTCID50/0.1 ml. For example, if we got an *n* number of IgTCID50/0.1 ml, it meant 0.1 ml sample solution brought a 50% morbidity of indicating cells when the sample solution was diluted into 10 to the *n* folds.

The viruses were originally purchased from ATCC. PRV and PPV were propagated in ST cell culture. EMCV and Sindbis virus were propagated in Vero cell culture. In brief, viruses were inoculated when cells reach confluent. The cell culture was maintained with 5% FBS in MEM, at 37°C and 5% CO_2_ atmosphere in an incubator. The cell culture was terminated after more than 90% of cells showed pathological change. The viruses were released after three cycles of a freeze-thaw process of the culture flask. The cell debris was removed by centrifuging at 3,000 rpm for 10 min. The supernatant containing viruses was collected and stored at −70°C for the following experiments.

Viruses were loaded on actual processed samples before the virus inactivation point designed to mimic the real circumstances. For the initial loading, amnions, chorions, or amnion/chorion complexes were immersed within viral solutions overnight at 2–8°C. Next, the concentrations of the viruses were detected and recorded as the initial loading. The virus-loaded samples were immersed within the 50 μmol/L RB working solution for 2 h. The virus loading was recorded and detected as the control. Then, samples were challenged with 0, 3, 5, or 7 J/cm^2^ UVB exposure in a UVB crosslinker (Spectronics, XL-1500B/FA), and the virus load was compared to the control to calculate the fold reduction of virus. For Co-60 irradiation, the amnion/chorion complex was challenged with 10 kGy, 15 kGy, or 17.5 kGy Co-60 irradiation to determine the reduction of the virus titer.

### Animal experiments

Animal experiments were performed by Shandong Xinbo Pharm. Ltd. A chronic wound model with an induced toxic level of reactive oxygen species (ROS) around the wound area was developed as previously described (Dhall et al., 2014; Dhall et al., 2018; Dhall et al., 2019). In brief, 20 min prior to creating 7 mm wounds, db/db mice were treated once intraperitoneally with 3-amino-1,2,4-triazole (Aldrich Chemistry, St. Louis, MO), at 1 g/kg body weight. Immediately post-injury, wounds were treated topically with mercaptosuccinic acid (MSA) (Sigma, St. Louis, MO), at 150 mg/kg body weight and covered with Tegaderm™ (3M, St. Paul, MN). The animals were anesthetized using 1% isoflurane prior to the wounding and put on a heating pad for 30 min to avoid post-surgical hypothermia after the operations. Within 14 days, wounds became chronic. These chronic wounds were treated weekly with topical applications of the first layer of either amnion/chorion complex or Mepitel^TM^ (Molnlycke Health Care, Finland) and the secondary wound dressing of Tegaderm, or Tegaderm alone as blank control. Animal weights and wound photos were collected weekly. The wound area was calculated using ImageJ. All animal procedures and animal care were performed in accordance with the Shandong Laboratory Animal Regulations. All animal procedures were approved by the Animal Welfare and Ethics Committee of Shandong Xinbo Pharm. Ltd.

### Histology

For histology tests, the samples were fixed with 4% paraformaldehyde for at least 24 h, embedded with paraffin, sliced, and subjected to HE, Verhoeff–Van Gieson, and Masson trichrome staining procedures using standard protocols.

### Statistics

The statistic calculation method was indicated at the end of the annotation of each figure or table.

## Results

### The screening of washing solutions and drying methods

It was reported that amnion and chorion tissues could be preserved by dehydration and the following sterilization steps for long-term storage without losing their tissue-repairing potency (Koob et al., 2013; Koob et al., 2014; Koob et al., 2015). Before the dehydrating steps, the tissues needed to be cleaned and washed. Biomaterials can be salted for preservation. Amnion and chorion tissues were reported to be washed in hypertonic saline solutions (Daniel et al., 2013). However, there was not any report about whether the action of salting-out would diminish the bioactive factors presented within amnion and chorion tissues. For the aforementioned reasons, we compared three rinsing solutions for the washing steps, that is, pure water, isotonic saline, and hypertonic saline. We also compared the dehydration methods of freeze-drying and air-drying. The evaluation marks were chosen as bFGF and HGF, according to the literature review and the high-throughput multicytokine-array screening results (Choi et al., 2009; Rodriguez-Ares et al., 2009; Koob et al., 2013; Koob et al., 2014; Koob et al., 2015).

**FIGURE 1:**
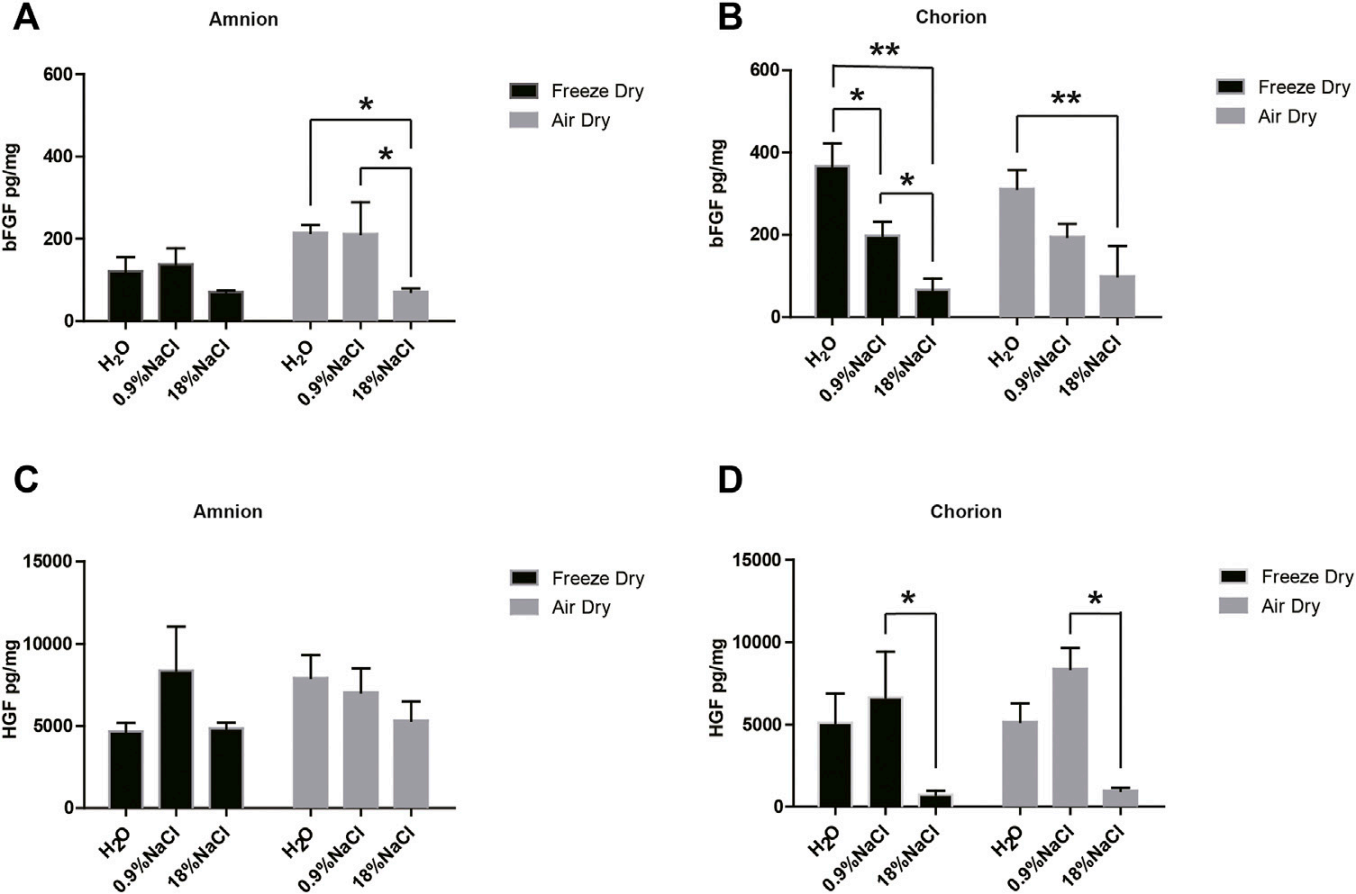
bFGF and HGF content of amnion and chorion after they were washed with different solutions and dried by different methods. Generally, hypertonic saline reduced the growth factor compared with normal saline. Compared with normal saline, **(A)** hypertonic saline in the washing step significantly reduced the bFGF content in amnion tissue following the air-dry step and **(B)** reduced the bFGF content in chorion tissue following the freeze-dry step. **(D)** Hypertonic saline also brings a statistically significant decline of HGF content in chorion tissues following either the air-dry or the freeze-dry procedure. The air-dry procedure maintained similar content of bFGF and HGF growth factors compared with the freeze-dry procedure. Data represent mean ± SD and *n* = 3 biological repeats for each group. *p < 0.05 and **p < 0.01. One-way ANOVA with Tukey’s *post hoc* test was used for the calculation.

Not a surprising result showed that hypertonic saline in the washing step has a general tendency of reducing the growth factors of bFGF and HGF, compared with normal saline or pure water (Figure 1). Specifically, compared with normal saline, hypertonic saline in the washing step significantly reduced the bFGF content in amnion tissue following the air-dry step and reduced the bFGF content in chorion tissue following the freeze-dry step. Hypertonic saline also brings a statistically significant decline of HGF content in chorion tissues following either the air-dry or the freeze-dry procedure. A growth factor multicytokine-array screening experiment confirmed the results, since normal saline persevered generally more growth factors related to cell proliferation in comparison with hypertonic saline in washing steps, although not in all the cases of detected growth factors (Table 1). As a result, we decided to choose normal saline as the washing reagent for the following experiments. Pure water was another option, but we found that the tissues swelled a lot and became fragile after keeping in pure water for a long period.

**TABLE 1:** Multicytokine-array screening results of the amnion/chorion complex derived from normal saline or hypertonic saline washing.

Growth factor type		Concentrations of the 0.9% NaCl group (pg/ml)	Concentrations of the 18% NaCl group (pg/ml)	Notes
Angiogenesis factors	LAP (TGF*β*1)	1,389.7	526.3	
	FGF-6	665.8	2,833.6	
	HGF	6,090.0	1,926.2	
	ANG-2	120.9	59.2	
	EG-VEGF	300.5	575.1	ED_50_ 1–4 μg/ml
Growth factors known effective for epithelial cells	IL-20	11,275.5	18,669.5	
	EGF	18.6	15.2	
Growth factors known effective for neural cells	Midkine	7,487.5	954.7	
	BDNF	177.9	21.8	
Growth factors known effective for fibroblasts	FGF-6	665.8	2,833.6	ED_50_ 0.1–0.3 ng/ml
	PDGF-BB	654.4	522.9	ED_50_ 1.5–6 ng/ml
	PDGF-AB	739.1	91.4	ED_50_ 1–3 ng/ml
	FGF-9	44.3	422.5	ED_50_ 1–5 ng/ml
	bFGF	983.2	11.5	ED_50_ 0.1–0.6 ng/ml
	PDGF-AA	358.6	213.5	ED_50_ 50–200 ng/ml
	TGFα	192.6	93.0	ED_50_ 0.1–0.4 ng/ml

ED_50_ was looked up from the website of R&D systems.

**FIGURE 2:**
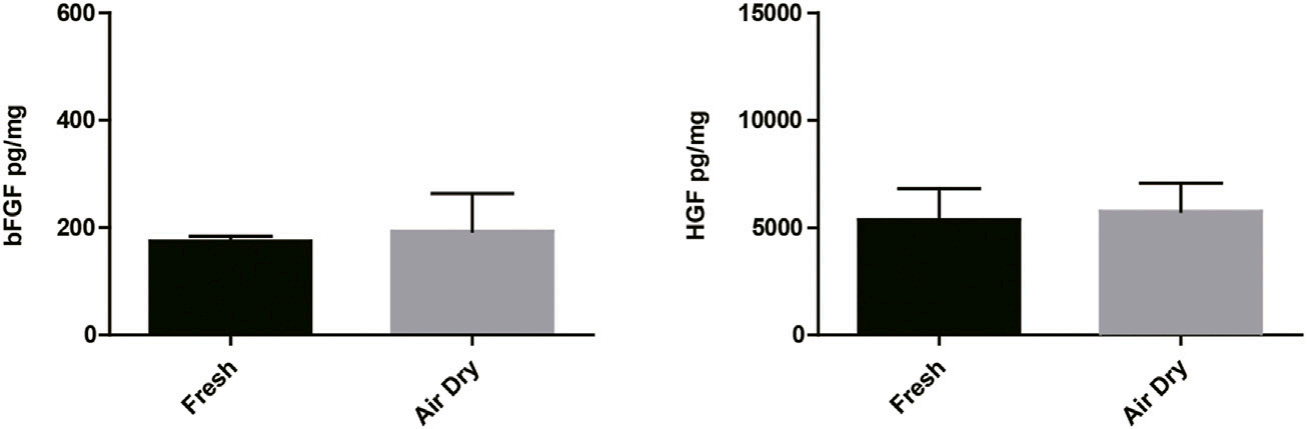
bFGF and HGF testing results of the air-dry-derived amnion/chorion complex and fresh tissues. The air-dry procedure maintained similar content of bFGF and HGF growth factors compared with the fresh tissues. Data represent mean ± SD and *n* = 3 biological repeats for each group. Student’s t-test was used for statistical analysis, and data were not significantly different for each group.

The air-dry procedure maintained similar content of bFGF and HGF growth factors compared with the freeze-dry procedure (Figure 1). We did not observe any obvious difference in the quantity of growth factors between air-dry and freeze-dry methods. In addition, air-dry preserved bFGF and HGF concentrations of the amnion/chorion complex similar to freshly isolated tissues (Figure 2). The freeze-dry derived samples were less translucent compared with the air-dry, which was supposed to be caused by the very small ice crystals formed during the freeze-dry course, and it may affect the appearance for ophthalmologic uses, so we decided to use air-dry as the dehydration method for the following experiments.

### Virus inactivation

**FIGURE 3:**
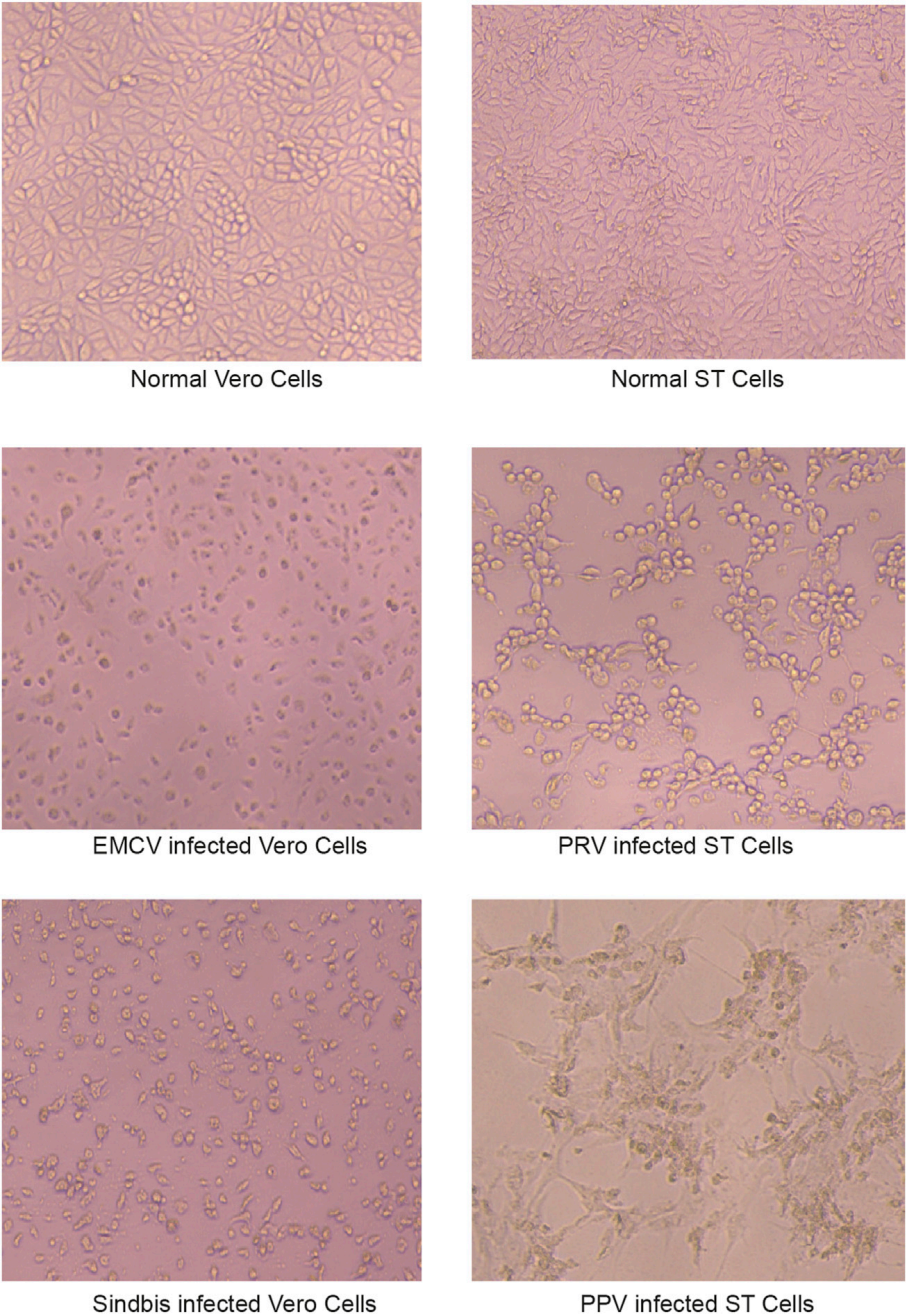
The indicating cells for virus concentration determination. ST cells were used as the indicating cells to calculate the reduction of PRV and PPV titer. Vero cells were used for Sindbis and EMCV titer.

**FIGURE 4:**
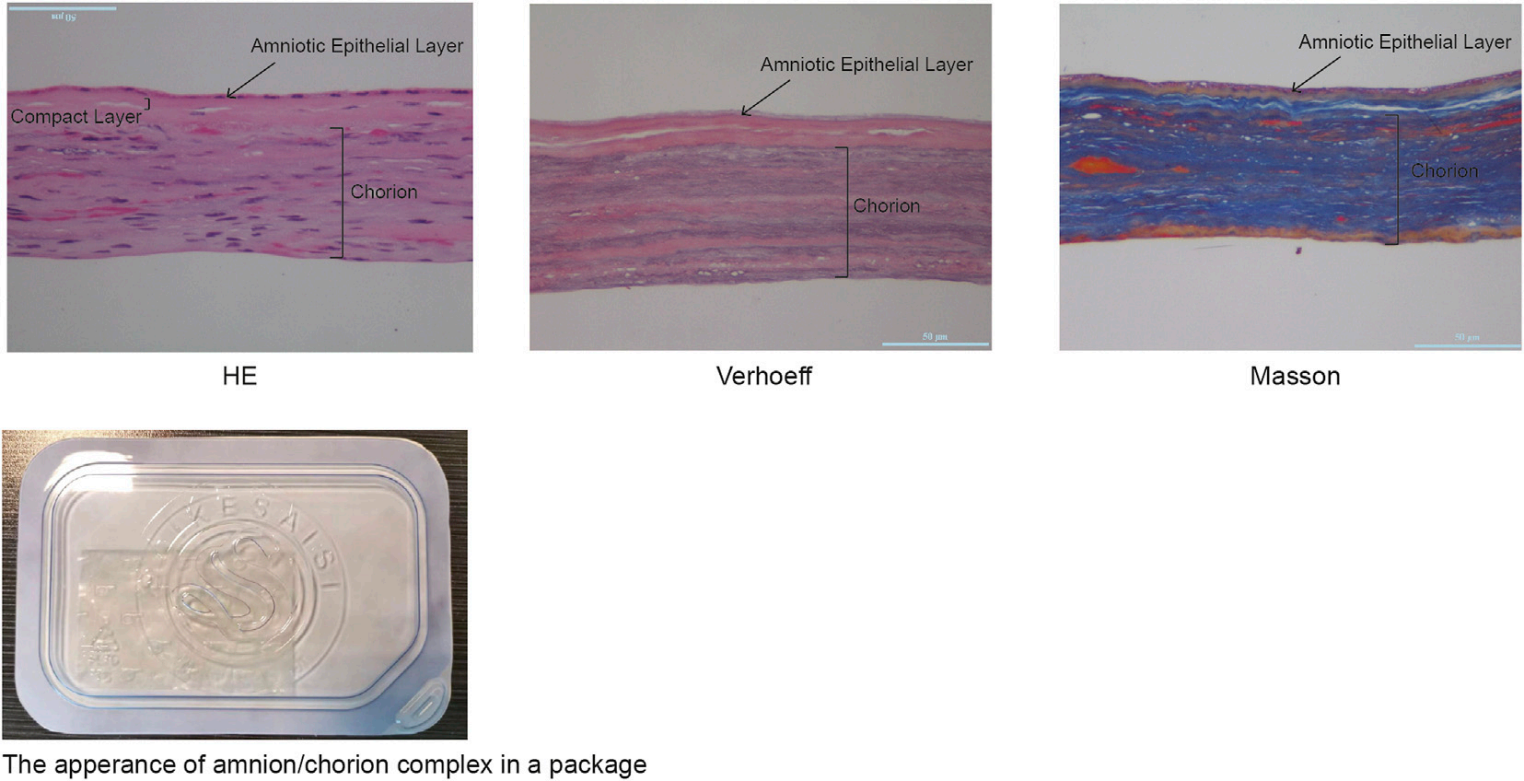
The histological analysis of the amnion/chorion complex. Histological analysis showed the amnion/chorion complex contained a multilayer structure.

According to the recommendations of the WHO, the virus inactivation process needs to achieve as high as a 10^6^-fold reduction of virus (Who, 2004). It would be preferable to use two inactivation procedures, the mechanism of which is complementary to each other for the manufacturing process, and each procedure capable of reducing 10^4^ folds of the virus titer. The amnion and chorion like other biomaterial products are usually sterilized using e-beam or Co-60 but high doses of irradiations would diminish the therapeutic capacity of biological products. Mirasle is a well-documented method for pathogen inactivation including viruses and is used for whole blood or blood components such as plasma and platelets (Martin et al., 2005; Schlenke, 2014; Keil et al., 2015; Cap et al., 2016). It employs RB, a photosensitizer that mediates selective damage to nucleic acids under the exposure of ultraviolet light. The process involves an oxygen-independent electron transfer process leading to the irreversible damage of nucleic acids, primarily on guanine residues, and the conversion of RB to its photoproduct lumichrome (LC), which otherwise would be reversible with UV itself (Marschner and Goodrich, 2011). We tested whether this RB-UV virus inactivation method will be useful on amnion and chorion biomaterials. The RB-UV method was modified from the Mirasle method to meet the local conditions. Four types of viruses, PRV, Sindbis virus, EMCV, and PPV were used as model viruses for the verification of the RB-UV method. Each represents one of the four types of viruses, DNA enveloped, RNA enveloped, DNA non-enveloped, and RNA non-enveloped. Standard *in vitro* limiting dilution assays were performed to determine the virus concentration and verify the viral reduction. Amnion or chorion tissue was loaded with model viruses and spread on a tray within a UVB crosslinker. After the inactivation process, tissues were homogenized. The virus concentrations of the supernatant were then determined using the indicating cells (Figure 3). The RB-UV method substantially reduced the infectious load of all the four types of viruses in our test systems, suggesting the potential to provide a significant level of protection against viruses and to close the window period that exists for screened viruses. As low as 3 J/cm^2^ energy of delivered UV eliminated all four types of viruses within the system (Tables 2, 3). We also tested the Co-60 irradiation as the method of viral inactivation for the amnion/chorion complex membrane. Data showed that, apart from the PPV virus, all the rest three types of viruses were very sensitive to Co-60 irradiation (Table 4). PPV required at least 15 kGy of irradiation dose whereas PRV, Sindbis virus, and EMCV were eliminated by as low as 10 kGy of irradiation (Table 4). Data were consistent with previous findings, DNA non-enveloped viruses, such as PPV and B16, were more resistant to Co-60 irradiation (Grieb et al., 2002). Our data suggest the RB-UV method can be a complementary method to the classic Co-60 irradiation for entirely removing the potential viruses within allogenic tissue grafts. Using the aforementioned preservation and sterilization method, we developed a sterilized amnion/chorion complex for the following animal experiments. In brief, amnion and chorion were washed using normal saline, immersed with RB, and exposed with 5 J/cm^2^ energy UV, amnion layered with chorion as a complex and air-dried, then sterilized with 17.5 kGy irradiation of Co-60. The histology analysis showed they contained multiple layers (Figure 4).

**TABLE 2:** Viral reduction of amnion (values in log) by UV-RB.

		Virus			
		PRV	Sindbis	EMCV	PPV
UVB irradiation (Joule)	0	1.44 ± 0.39	0.92 ± 0.39	1.31 ± 0.13	0.71 ± 0.33
	3	≥5.06	≥5.40	≥6.15	≥5.17
	4	≥5.06	≥5.40	≥6.15	≥5.17
	5	≥5.06	≥5.40	≥6.15	≥5.17
	6	≥5.06	≥5.40	≥6.15	≥5.17

Data represent mean ± SD, and *n* = 3 biological repeats.

**TABLE 3:** Viral reduction of chorion (values in log) by UV-RB.

		Virus			
		PRV	Sindbis	EMCV	PPV
UVB irradiation (Joule)	0	1.94 ± 0.22	1.37 ± 0.36	1.19 ± 0.13	1.25 ± 0.18
	4	≥6.14	≥6.25	≥6.84	≥5.40
	5	≥6.14	≥6.25	≥6.84	≥5.40
	6	≥6.14	≥6.25	≥6.84	≥5.40

Data represent mean ± SD, and *n* = 3 biological repeats.

**TABLE 4:** Viral reduction of the amnion/chorion complex (values in log) by Co-60 irradiation.

		Virus			
		PRV	Sindbis	EMCV	PPV
Co-60 irradiation (kGy)	0	≥3.98	≥3.54	0.89 ± 0.30	0.77 ± 0.39
	10	≥4.77	≥4.73	≥4.75	3.07 ± 0.19
	15	≥4.77	≥4.73	≥4.75	≥5.17
	17.5	≥4.77	≥4.73	≥4.75	≥5.17

Data represent mean ± SD, and *n* = 3 biological repeats.

### Skin repair tests

**FIGURE 5:**
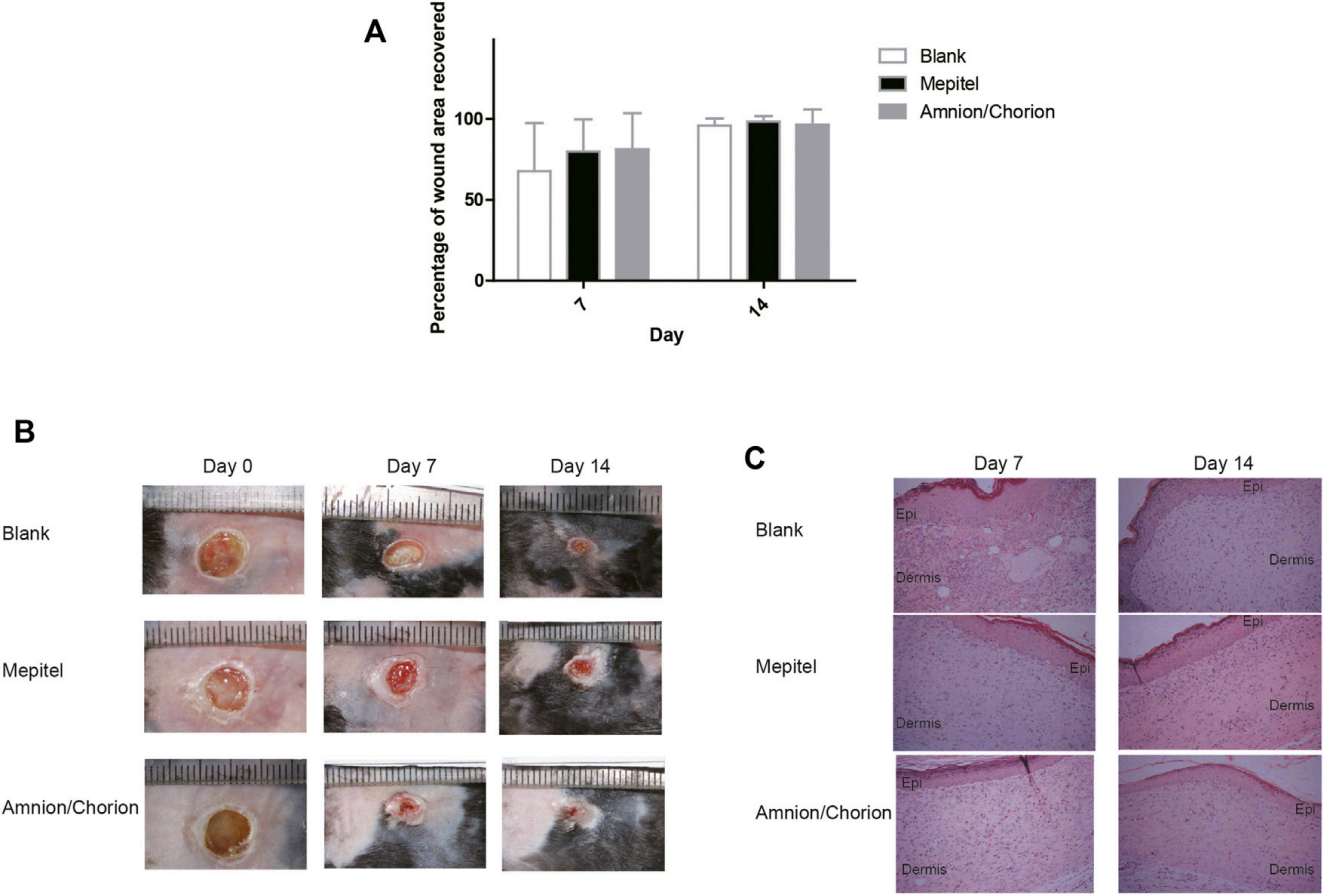
The skin repair capacity of amnion/chorion complex. **(A)** There was a tendency of accelerated recovery in terms of wound area for the amnion/chorion complex graft group compared with the blank control on day 7, although the data was not statistically different. Data represent mean ± SD and *n* = 20 for day 7 and *n* = 10 for day 14. One-way ANOVA with Tukey’s *post hoc* test was used for the calculation. **(B)** Representative photos of the wound recovery of the animal model. **(C)** Histology results of the recovered wound area. There was no apparent difference between each group.

There are several animal models often used for diabetic wound analysis, including the STZ-induced rat model, the splinted wound model, and the transgenic db/db mouse model (Dhall et al., 2014; Park et al., 2014; Yu et al., 2017; Dhall et al., 2018; Dhall et al., 2019). We used the established db/db diabetic mouse model having an elevated toxic level of reactive oxygen species (ROS) around the wound area, the rate of wound closure of which is significantly delayed in comparison to non-diabetic animals (Dhall et al., 2014; Dhall et al., 2018; Dhall et al., 2019). To mimic the real circumstances, the amnion/chorion complex and controls were applied 2 weeks after the initial wounding. The application date was recorded as day 0. We could observe a tendency of accelerated recovery in terms of wound area for the amnion/chorion complex graft group compared with the blank control on day 7, although the data was not statistically different (Figure 5). The amnion/chorion complex showed similar wound repair capacity to Mepitel, a commercial wound contact layer containing adhesive silicone. However, it was statistically significant in terms of the number of fully recovered animals on day 7 and on day 14, and the data favored the amnion/chorion group compared with the blank control (Table 5). There was no obvious difference in the histological examination of each group.

**TABLE 5:** Number of animals completely recovered.

		Blank	Mepitel	Amnion/chorion
Day 7	Completely recovered	1	5	6*
	Not fully recovered	19	15	14
Day 14	Completely recovered	2	7*	7*
	Not fully recovered	8	3	3

More animals were fully recovered on day 7 and on day 14 for the amnion/chorion complex group, compared with blank control. Data represent mean ± SD and *n* = 20 for day 7 and *n* = 10 for day 14. *p < 0.05; Pearson’s chi-square test was used for the calculation.

## Discussion

The amnion and chorion like other biomaterial products are usually sterilized using e-beam or Co-60. However, high doses of irradiations would diminish the therapeutic capacity of biological products. It is recommended to use at least two inactivation procedures, and the mechanism of them was complementary to each other. We investigated whether the RB-UV method was capable of removing the viral load within amnion or chorion grafts to a level substantially acceptable and whether it can be used as a regular virus inactivation approach, considering amnion is a relatively translucent tissue and chorion is also semi-translucent after the removal of the residue of decidual layer tissues. The method of UV together with light-sensitive Riboflavin (RB-UV) has been used for virus inactivation of whole blood or blood components to close the window period of virus screening. The method is known for its biosafety and nonharms for whole blood and blood components (Marschner and Goodrich, 2011). But there wasn’t any report on whether it can work on biomaterials. To test the hypothesis, standard *in vitro* limiting dilution assays were employed to verify the virus inactivation method. We found that as low as 3 J/cm^2^ energy of UV was able to reduce the virus load by 10^5^ folds for all four types of model viruses loaded on the amnion and the chorion, indicating the RB-UV method was effective to inactivate DNA, RNA, enveloped and non-enveloped viruses. Our data showed PPV was less sensitive to Co-60 irradiation compared with the other three types of model viruses. The results were consistent with previous reports, that DNA non-enveloped viruses such as PPV and B16 were resistant to Co-60 irradiation. As a result, RB-UV is complementary to the classic Co-60 irradiation for virus inactivation to reduce the dose of Co-60 irradiation. When the two methods, RB-UV and Co-60 combined, a very low amount of irradiation was required to meet the standard of 10^6^ folds reduction of virus titer. RB-UV method has additional merits since it is an easy approach and can be performed in every laboratory with basic experiment conditions. In addition, the virus inactivation method usually needs to be verified for the development of any novel biological product. The RB-UV method can be verified domestically rather than transported to the remote and restricted Co-60 site, which is especially advantageous for verifying the inactivation of some dangerous viruses such as HIV.

The commonly using virus inactivation technologies include the treatment with gamma irradiation, heat or UV-radiation; the treatment with photoactive compounds such as methylene blue, psoralens, or 1.5-iodonaphtyl azide; the treatment with ionic or non-ionic detergents such as Tween-20/Tween-80, Triton X-100 or sodium dodecyl sulfate and with solvents such as alcohol and acetone (Elveborg et al., 2022). The combination of simple techniques such as heat, UV-radiation, or detergents are often sufficient for virus inactivation but the efficiency depends on the infectious pathogens and the nature of the biomedical products. The merits of the RB-UV method presented by the current report mainly rely on its low energy of irradiation and its free of harmful additional reagents, since the safety of riboflavin is fully understood (Reddy et al., 2008; Hellstern and Solheim, 2011; Marschner and Goodrich, 2011). In contrast, some of current techniques have their disadvantages. Solvent (tri-n-butyl phosphate, TnBP) and detergent (Triton X-100) methods were developed to inactivate enveloped viruses in plasma protein preparations; however, a resin needs to be used for the removal of the solvent and detergent after the performed solvent/detergent (S/D) inactivation procedures (Hellstern and Solheim, 2011). Pasteurization requires a temperature of 60°C. Low pH virus inactivation method may be a denaturing condition for certain growth factors. Using methylene blue may result in the color change of the biomaterial.

The current report mainly presents data about the virus inactivation. An obvious fact is that, in addition to viruses, the removal and inactivation of other pathogens, bacterial in particular, needs to be taken into consideration for the development of a biomaterial-based medical device. For terminal sterilization, the chosen dose of Co-60 is dependent on the upper limit of bioburden borne by the products and the following verification tests. The average bacterial bioburden of the amnion/chorion biomaterial was controlled less than 9 after the process of the washing steps and the RB-UV method, which requires a Co-60 dose of 17.5 kGy to achieve a sterility assurance level (SAL) of 10^–6^. The dose was determined and verified according to a standard method (Ansi/Aami/Iso, 2013). The bioburden can be further reduced by improving the manufacturing environment and optimizing the standard operation procedures.

We also analyzed whether the growth factor content of amnion and chorion was affected by the washing reagents. Amnion has been reported to contain numerous growth factors and MMPs, including bFGF, HGF, KGF, EGF, and PDGF-BB. We found that, compared with normal saline, hypertonic saline substantially reduced the number of growth factors, using bFGF and HGF as the marker. We also compared the freeze-dry and air-dry procedures, amnion/chorion tissues derived from both drying methods reserved similar growth factor contents. The main contaminating maternal cells in chorion and amnion are usually blood cells. The vigorous washing steps were required to remove most of the blood cells within the tissue, although there were still rare blood cells sparsely residing within the chorion tissue with minimal risks. The washing process can be simply monitored and verified by urine occult blood test strips to minimize the risks. In addition, there were residues of degenerated maternity-origin decidual layer tissues attached to the chorion layer, although there is no report on the immunogenicity of chorion tissues despite their broad clinical applications.

Using the washing, drying, and viral inactivation methods described earlier, we made an amnion/chorion complex graft and tested the tissue-repairing capacity of it. We compared the amnion/chorion complex, Mepitel, and the blank control on a diabetic wound model. The application of the amnion/chorion complex and Mepitel has a tendency of accelerated recovery in terms of wound area compared with control although data were not statistically significant. The data favored the amnion/chorion group compared with the blank control on day 7 and day 14 in terms of the number of fully recovered animals.

## Conclusion

We herein provide an RB-UV method for virus inactivation of amnion and chorion tissues. The present article also describes a way of washing and drying of amnion and chorion tissues, which greatly retained the growth factors.

## Data Availability

The raw data supporting the conclusion of this article will be made available by the authors, without undue reservation.
