# Corrigendum: A minimally manipulated preservation and virus inactivation method for amnion/chorion

**DOI:** 10.3389/fbioe.2023.1210771

**Published:** 2023-05-16

**Authors:** Shang Zhang, Lichang Gao, Pin Wang, Yuyan Ma, Xiaoliang Wang, Jie Wen, Yu Cheng, Changlin Liu, Chunxia Zhang, Changfeng Liu, Yongli Yan, Chengru Zhao

**Affiliations:** ^1^ Success Bio-Tech Co., Ltd., Biomedical Material Engineering Laboratory of Shandong Province, Jinan, China; ^2^ Department of Gynecology and Obstetrics, Qilu Hospital, Cheeloo College of Medicine, Shandong University, Jinan, China; ^3^ Liangchen Biotechnology (Suzhou) Co., Ltd., Suzhou, China

**Keywords:** diabetic foot ulcer (DFU), chronic wound, animal model, amnion, virus inactivation, growth factor (GF)

In the published article, there was an error in Table 3 as published. A duplicate data of **Table 2** was accidently provided for Table 3. The corrected Table 3 and its caption “Viral reduction of chorion (values in log) by UV-RB” appear below.

**TABLE 3:** Viral reduction of chorion (values in log) by UV-RB.

		Virus			
		PRV	Sindbis	EMCV	PPV
UVB irradiation (Joule)	0	1.94 ± 0.22	1.37 ± 0.36	1.19 ± 0.13	1.25 ± 0.18
	4	≥6.14	≥6.25	≥6.84	≥5.40
	5	≥6.14	≥6.25	≥6.84	≥5.40
	6	≥6.14	≥6.25	≥6.84	≥5.40

Data represent mean ± SD, and *n* = 3 biological repeats.

The authors apologize for this error and state that this does not change the scientific conclusions of the article in any way. The original article has been updated.

